# Supplementary material for: Synthesis and Photophysical Properties of a Series of Dimeric Indium Quinolinates
Source: Molecules. 2020 Dec 23;26(1):34. doi: 10.3390/molecules26010034 (PMC7793487; doi:10.3390/molecules26010034)
Supplement: Supplementary file 1 [file molecules-26-00034-s001.pdf]

Supplementary Materials

Synthesis and Photophysical Properties of a Series of Dimeric Indium Quinolinates

Sang Woo Kwak,[1,†] Ju Hyun Hong,[2,†] Sang Hoon Lee,[1] Min Kim,[1] Yongseog Chung,[1] Kang Mun Lee,[2,*] Youngjo Kim,[1,*] Myung Hwan Park[3,*]

[1] Department of Chemistry, Chungbuk National University, Cheongju 28644, Republic of Korea, ykim@chungbuk.ac.kr (Y.K.)

[2] Department of Chemistry, Institute for Molecular Science and Fusion Technology, Kangwon National University, Chuncheon 24341, Republic of Korea, kangmunlee@kangwon.ac.kr (K.M.L.)

[3] Department of Chemistry Education, Chungbuk National University, Cheongju 28644, Republic of Korea, mhpark98@chungbuk.ac.kr (M.H.P.)

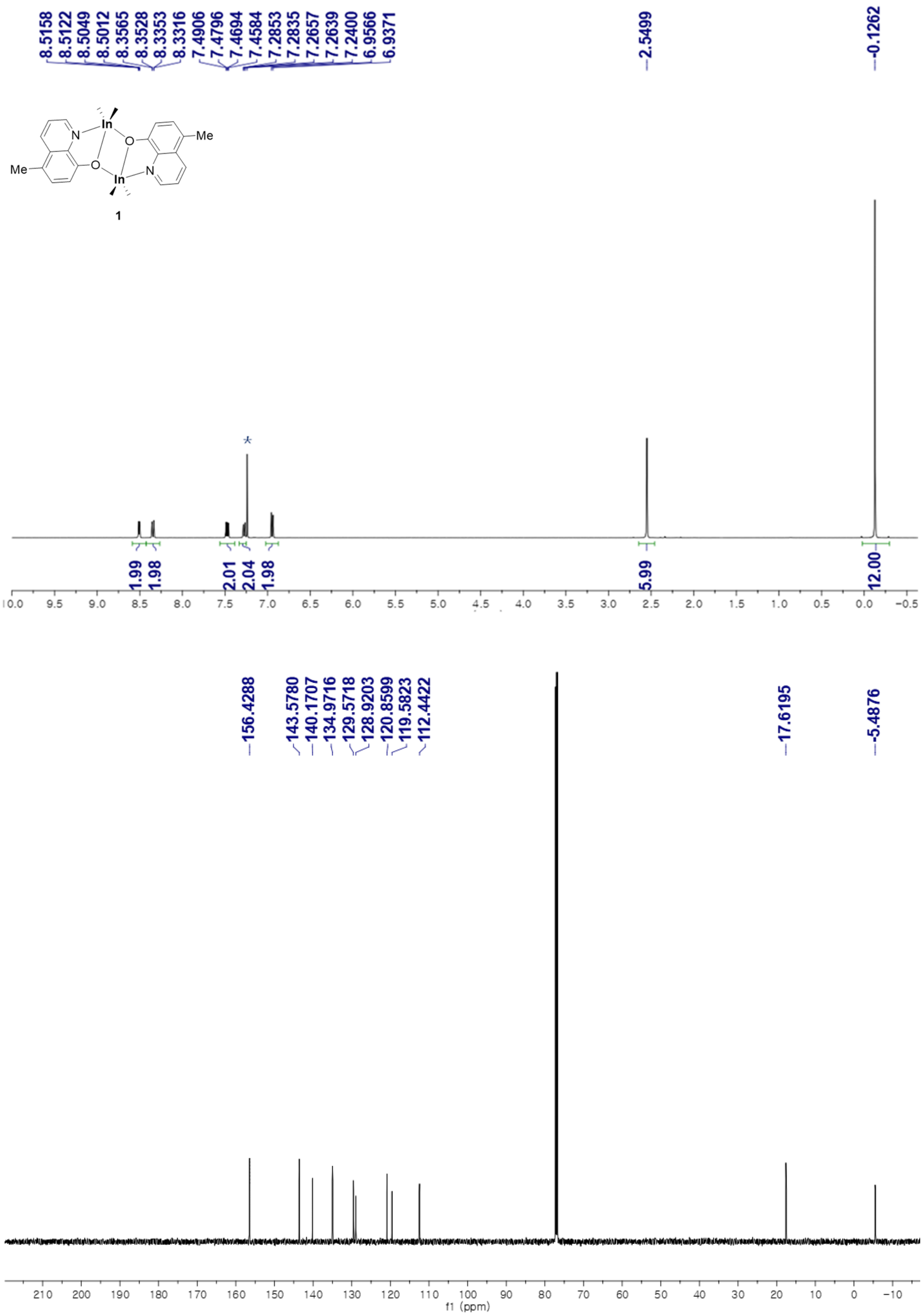

Figure S1. ^{1}H (top) and $^{13}C\{^{1}H\}$ (bottom) NMR spectra of **1** (*from residual $CHCl_3$ in $CDCl_3$).

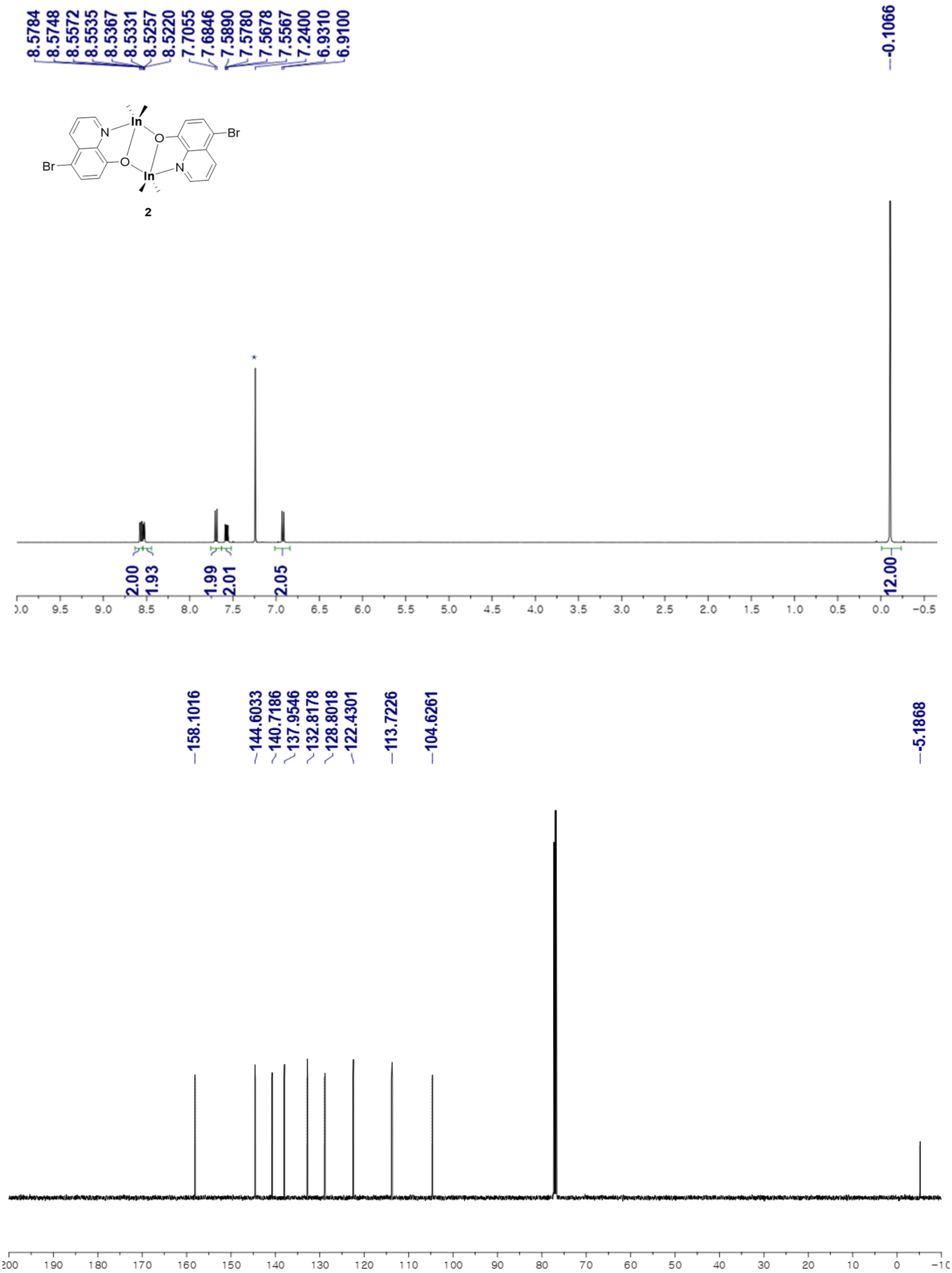

Figure S2. ^{1}H (top) and $^{13}C\{^{1}H\}$ (bottom) NMR spectra of **2** (*from residual $CHCl_3$ in $CDCl_3$).

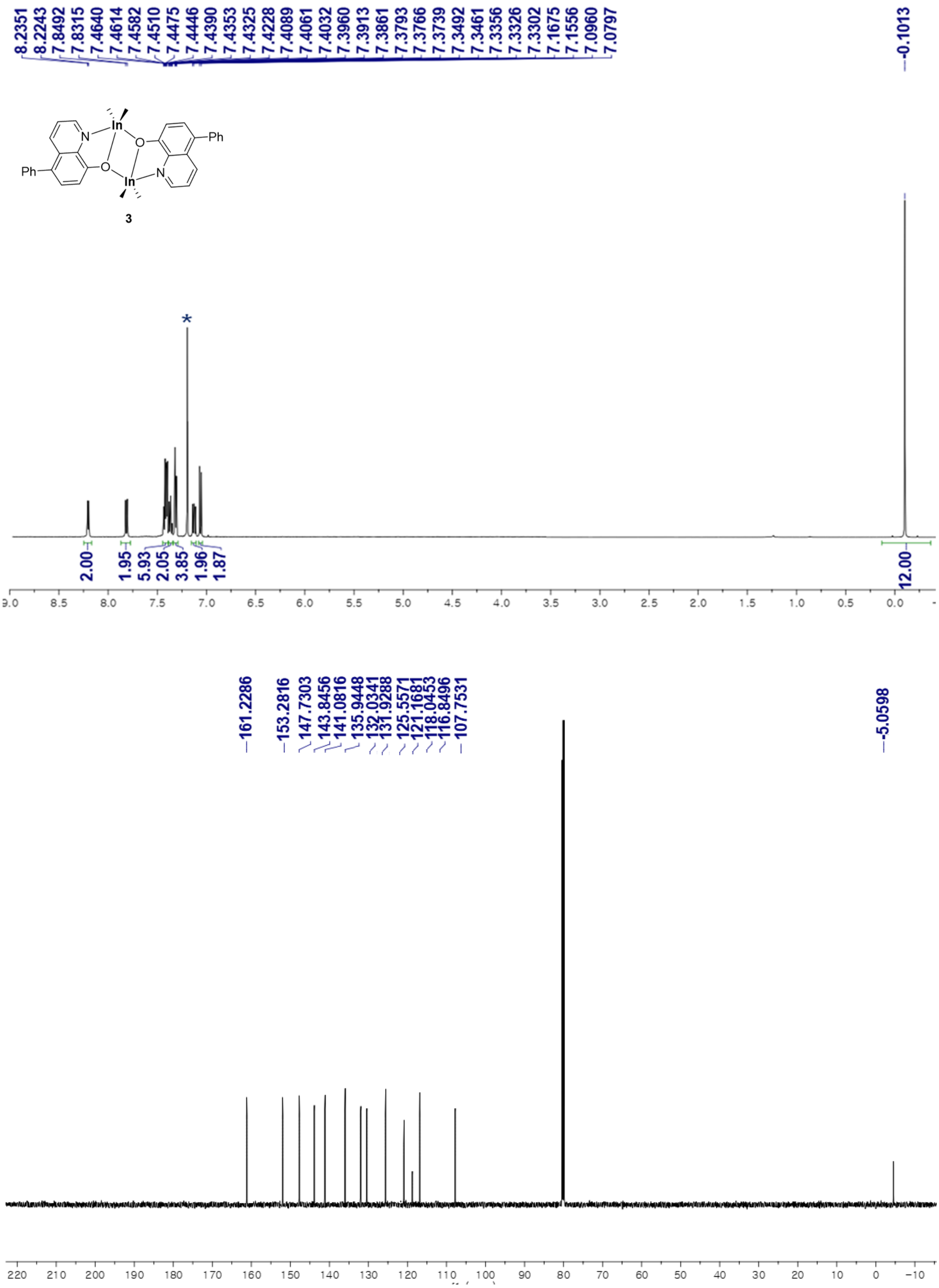

Figure S3. ^{1}H (top) and $^{13}C\{^{1}H\}$ (bottom) NMR spectra of **3** (*from residual $CHCl_3$ in $CDCl_3$).

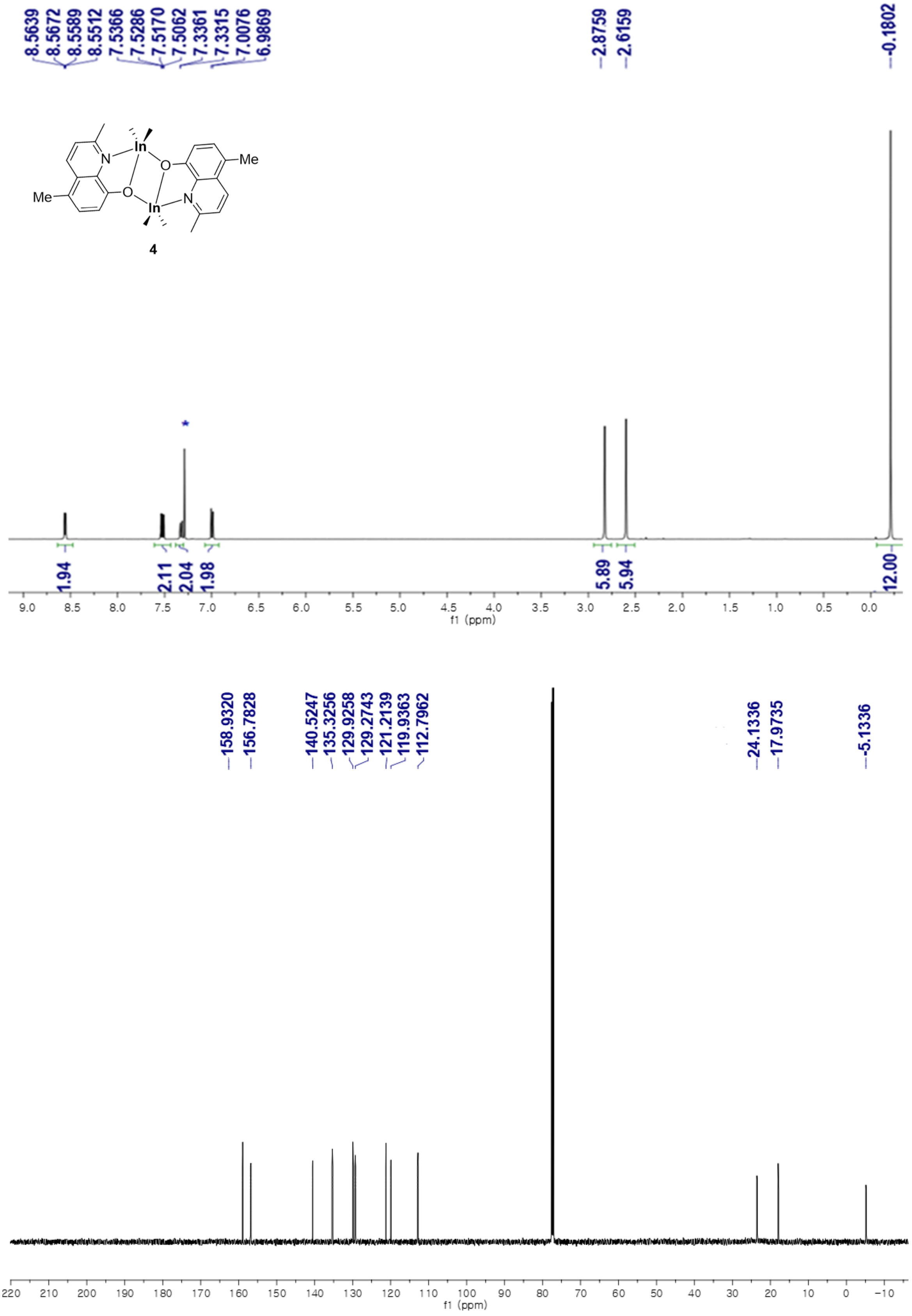

Figure S4. ^{1}H (top) and $^{13}C\{^{1}H\}$ (bottom) NMR spectra of **4** (*from residual $CHCl_3$ in $CDCl_3$).

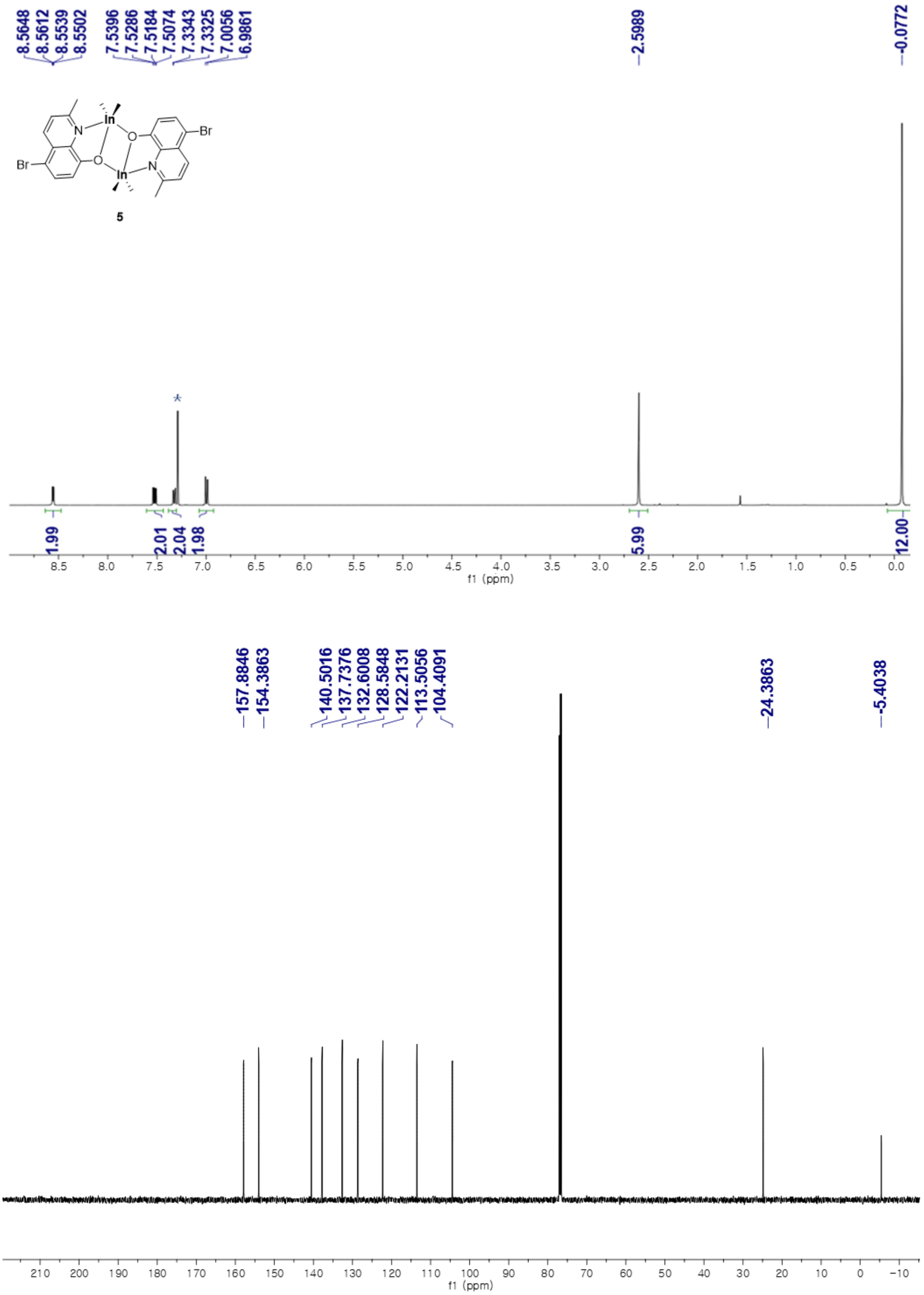

Figure S5. ^{1}H (top) and $^{13}C\{^{1}H\}$ (bottom) NMR spectra of **5** (*from residual $CHCl_3$ in $CDCl_3$).

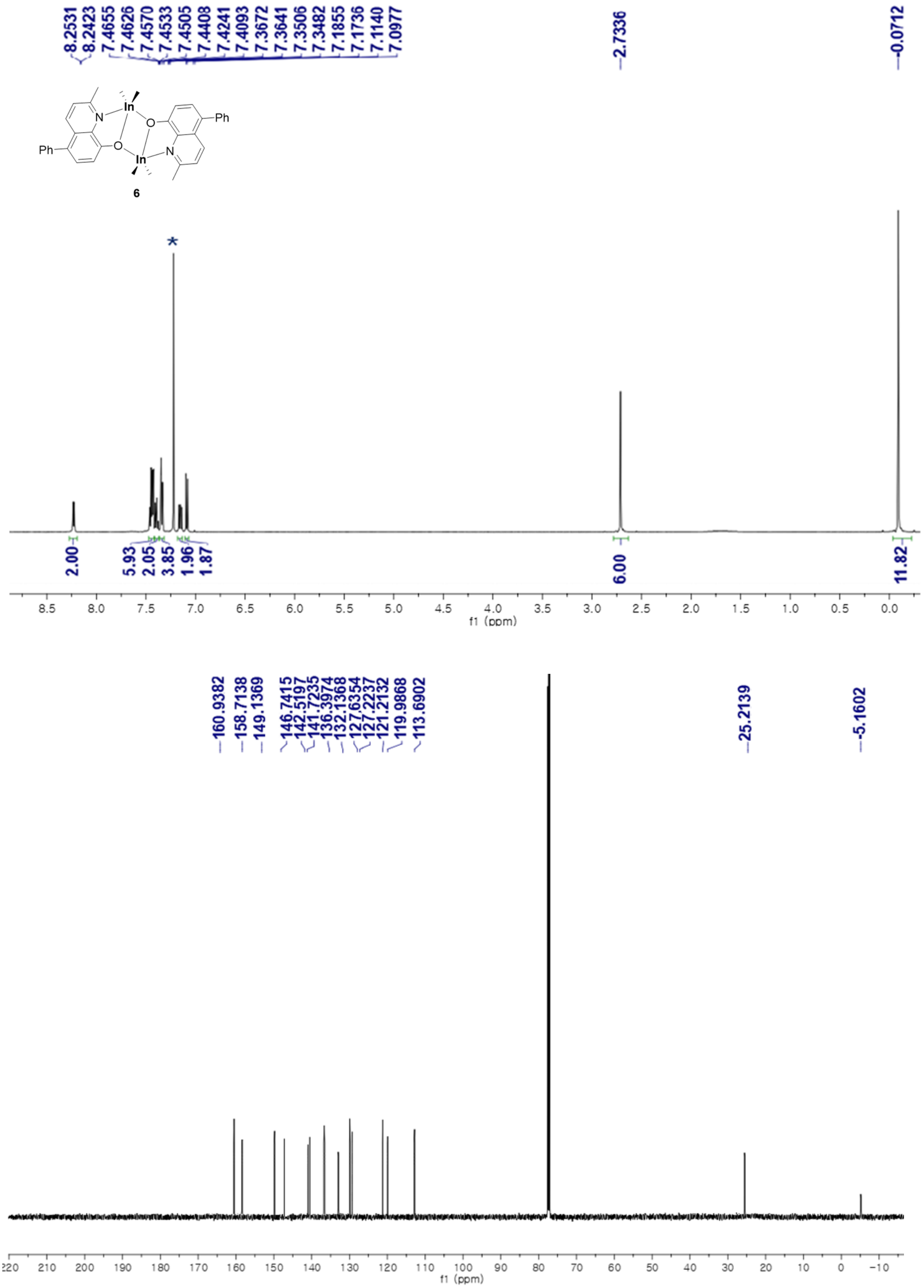

Figure S6. ^{1}H (top) and $^{13}C\{^{1}H\}$ (bottom) NMR spectra of **6** (*from residual $CHCl_3$ in $CDCl_3$).

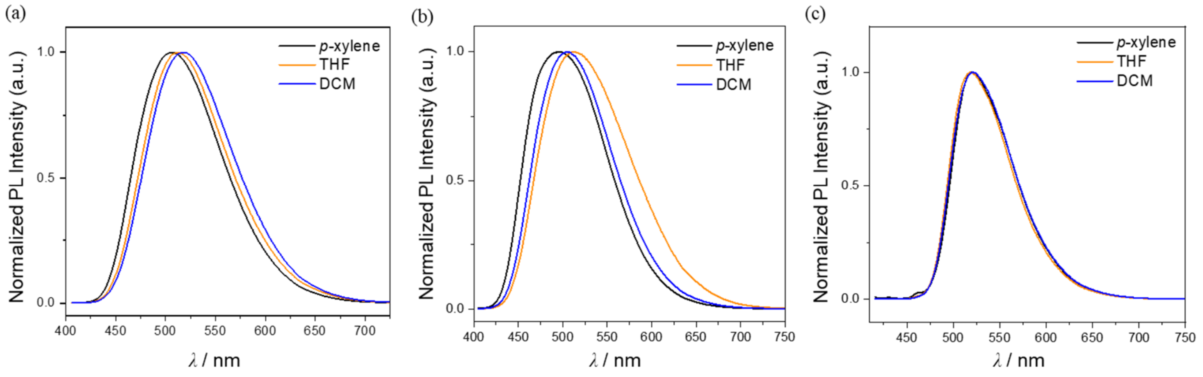

Figure S7. PL spectra of (a) **1**, (b) **2**, and (c) **3** in various organic solvents (50 μM) at 298 K.

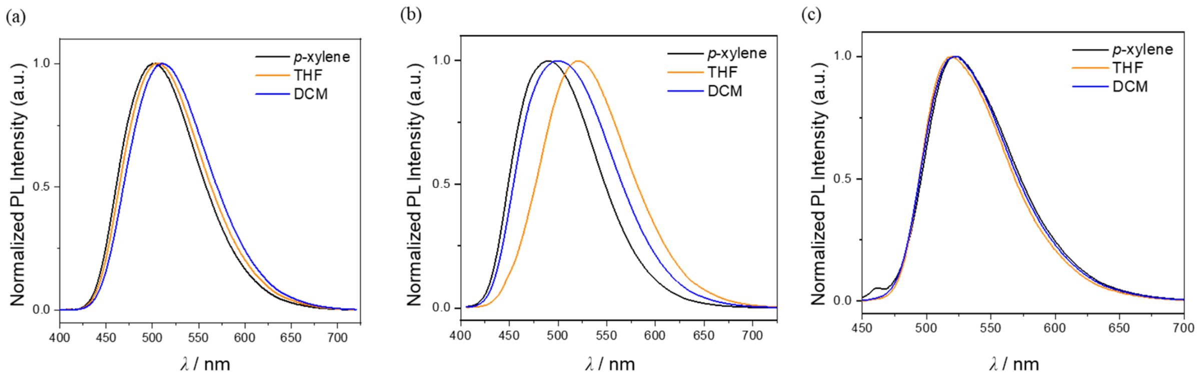

Figure S8. PL spectra of (a) **4**, (b) **5**, and (c) **6** in various organic solvents (50 μM) at 298 K.

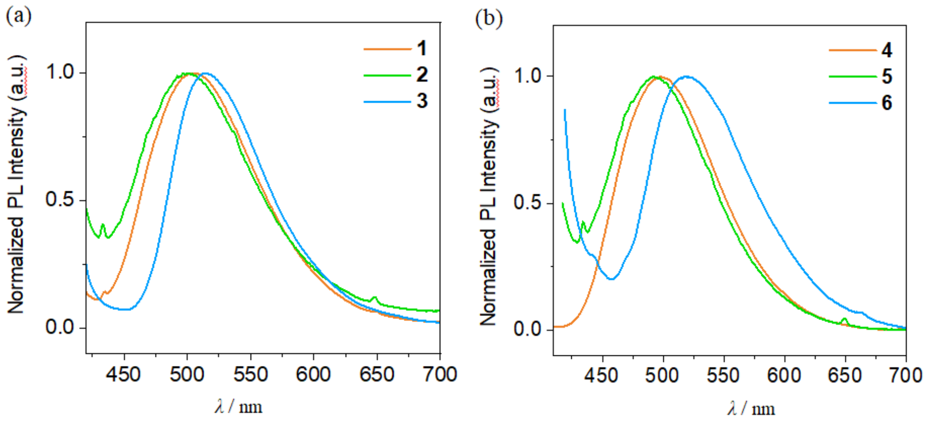

Figure S9. PL spectra of (a) **Inq**-based and (b) **InMq**-based complexes in film state (10 wt% doped on PMMA).

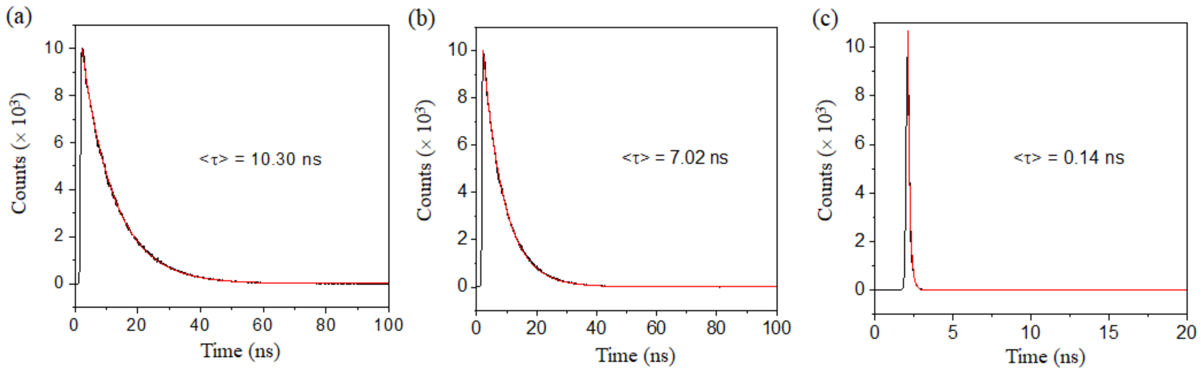

Figure S10. Emission decay curves for (a) **1**, (b) **2**, and (c) **3** in THF (50 μM). The red-line corresponds to the single-exponential fitting curves ($R^2 = 0.9995$ for **1,** 0.9994 for **2**, and 0.9905 for **3**).

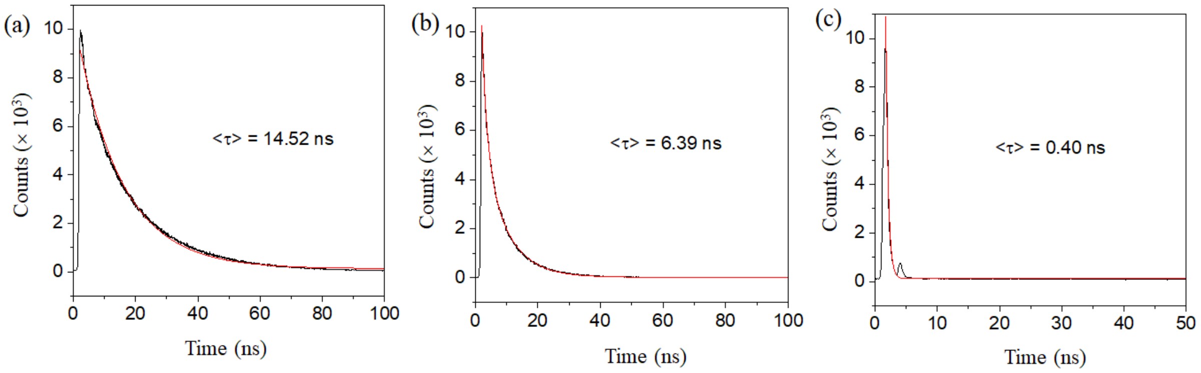

Figure S11. Emission decay curves for (a) **1**, (b) **2**, and (c) **3** in film (10 wt% doped on PMMA) at 298 K. The red-line corresponds to the single-exponential fitting curves ($R^2 = 0.9964$ for **1,** 0.9993 for **2**, and 0.9855 for **3**).

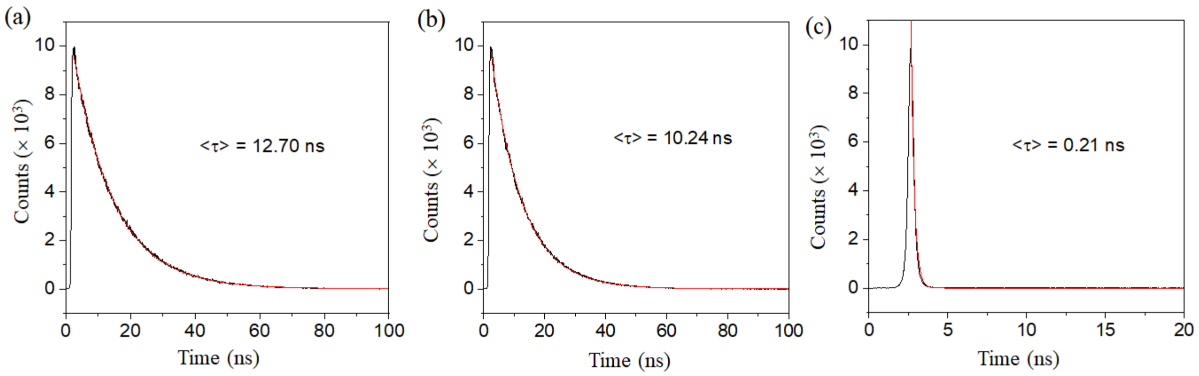

Figure S12. Emission decay curves for (a) **4**, (b) **5**, and (c) **6** in THF (50 μM). The red-line corresponds to the single-exponential fitting curves (R^2 = 0.9994 for **4,** 0.9995 for **5**, and 0.9896 for **6**).

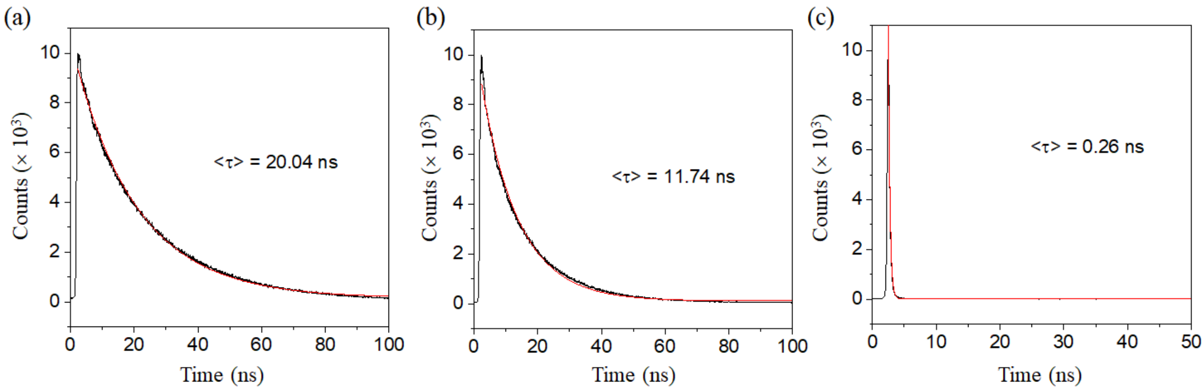

Figure S13. Emission decay curves for (a) **4**, (b) **5**, and (c) **6** in film (10 wt% doped on PMMA) at 298 K. The red-line corresponds to the single-exponential fitting curves (R^2 = 0.9981 for **4,** 0.9963 for **5**, and 0.9938 for **6**).
